# Our Calling

**Published:** 1881-09

**Authors:** F. Wm. Schwan

**Affiliations:** Poplar, O.


### ARTICLE V.

OUR CALLING. An Anniversary Address, delivered before the Seneca County (Ohio) Medical Society, August, 1880. By F. WM. SCHWAN, M.D., Poplar, O.

All human knowledge, save that which was strictly essential to the physical wants of man, has had a very obscure and primitive origin; and in every department of science, literature and art, we observe the same varying struggle for existence and progressive development. The conditions and relations of men have called forth the various kinds of knowledge that has sprung up, flourished and decayed, as their several wants have been supplied, or the necessity for them subsided.

Primitive man possessed but few attributes and wants, and these were only such as addressed themselves more especially to their physical understanding, prominent among which was no doubt the avoidance of bodily pain, and a dread of death. But as society advanced these conditions were more fully recognized and appreciated, and methods devised for relief. What these methods were will ever remain an impenetrable mystery, but tradition undeniably points out that pre-historic man possessed some crude means to alleviate suffering.

Passing from conjecture to proof, we find it asserted in the immortal Iliad, that at the siege of Troy, 1,100 years before the Christian era, the healing art had made considerable progress. Of course no corps of skilled surgeons then existed, but individual personages were frequently called upon to minister to the suffering, Agamemnon was called from the ranks of the besieging host to suck the poisoned wounds of Meneleos and Philoktel, and apply soothing salves. Achilles appears to have been especially qualified in the healing art, which he acquired from the Centaur Chiron. Patrokles had also become celebrated as a physician, and when Eurypelos was struck by an arrow, he removed the missile and dressed the wound with a soothing and healing herb, the virtues of which he had learned from Achilles, and which botany informs us to have been the *Achillea Millefolium*, or Yarrow.

Among some of the nations of antiquity it was customary to delegate persons who claimed to possess especial knowledge of the healing art to minister to the wants of the afflicted, and in Asia, Egypt, Assyria and Greece it was the practice to place the invalids on the street corners of the cities, with a placard fastened to them describing their symptoms and asking advice from any passer-by who may have been similarly affected. In some countries the people firmly believed that any derangement of the vital functions was a wrathful visitation of some deity, and the natural manifestation of disease was regarded as an evidence of displeasure of the offended spirit; and this belief being so indelibly rooted among the superstitious populace it became an easy matter for mountebanks and frauds to announce possession of supernatural powers, and by the practice of mysterious incantations and jugglery inspire faith, and proclaim themselves especially ordained as mediators between the invalid and the deity. If, perchance, the poor sufferer failed to receive the desired relief, it was always attributed to the want of belief on the part of the invalid.

In India and Egypt, the most cultured peoples of antiquity, we find invalids making pilgrimages to the sacred temples of Osiris, Isis, and Serapis. Here, also, we find mediators clothed in sacred vestments interposing for the relief of the pilgrims, before some special deity that was thought to preside over some particular part of the body, and, if some of our modern specialists could take a flight to ancient Memphis or Thebes they would be surprised to find how little they know of the manifold subdivisions into which the human form could be divided for treatment. From among the Brahmins of India, and Levites of Israel, sprang the lawgivers, physicians, priests and mediators; and it will be recollected that Moses, possessing not only the qualification of a lawgiver, but also that of a physician in an eminent degree, was enabled to give to his people a complete code of political ethics and laws of life, which being divested of their assumed sacredness, not only challenge our admiration but command our respect for their correctness. Solomon, also, gave to the Jews a system of "Laws of Health," and advised the priests to teach the treatment of disease by natural means, but those sacred officials resisted the innovation and had the tablets burned.

In Greece—the cradle of philosophy, of science, of literature and of art—the first grand forward stride in rational medicine was made, and in temples especially erected for the purpose, the god Asklepias and his daughters Hygeia and Pavakoia presided, and restored the invalids by teaching the observance of natural laws, based upon natural phenomena, and the application of practical hygiene in treatment.

The temples of Epidorus were also noted resorts for invalids, and were simply hygienic establishments from which the mysticism of antiquity had not been fully banished.

When Greece had arrived at the zenith of her glory; when Pericles directed her administration; when Herodotus enriched her literature with the splendor of his genius and Phidias inspired her art with the grandeur of his conceptions, when Plato and Socrates electrified mankind with the brilliancy of their attainments in metaphysics, medicine was yet a very little, sickly, weakly babe. But a nation that could bring forth such intellectual giants could not long remain in mental bondage. and gradually, almost imperceptibly, did the gods and goddesses lose their prestige, and, in the time of Aristophanes, we already find a numerous sect, known as Alklepias, who made a practice of observing disease and prescribing remedies; they also transferred the healing art from the sacredness of the temple to the private dwelling, and divested it of the mystery and jugglery that had characterized it from the beginning of the world. About this time was also established the noted gymnasii, in which were taught the observance of the laws of nature as well as the attainment of the highest physical development. Philosophy, also, was a mighty factor in shaping and moulding the minds of men, and when the great father of medicine, Hippocrates, came upon the stage of action, the world was not unprepared to welcome him. The attainments of this extraordinary man border on the marvelous, and his success was so great that his services were called into requisition not only in his own province but also in Macedonia, Thrace, Athens, Thessaly and Asia Minor. He accomplished a remarkable cure on the Macedonian king, Perdikkos, and restored to health Demokritos, of Abdera, for which he was offered ten talents of gold (nearly $12,000), but refused the compensation, claiming that the acquaintance of the renowned philosopher and savan richly compensated him. He was also called by Artaxerxes to Persia, which call he refused. With the advent of Hippocrates the calling of the physician assumed character, and the practice of medicine became a distinct branch of knowledge. But Greece had well nigh fulfilled her destiny in the category of nations, and her glory began to decline, and with it were scattered all her brilliant achievements —some to be lost forever, while others were rehabilitated in other lands. From Greece the new art was transferred mainly to Egypt, where the great Ptolemys exercised a patronizing care, and for 300 years new discoveries were constantly being added.

With the decline of Hellenic grandeur Rome became a resort for many of the followers of Hippocrates, but the ignorant populace received them with suspicion and ridicule, and finally drove them entirely out of the country. From this time on until the advent of Julius Cæsar, Rome contented herself with her soothsayers, her jugglers and her sybillic writers, and all there was of the healing art was in the hands of an ignorant and superstitious priestcraft, who created a special deity for every disease, prominent among which were the goddesses Febris and Cloacina, also a famous one named Scabies, from which we conclude that the holy fathers were familiar with the itch. Cæsar became a patron of medicine and gave it every encouragement, and by the efforts of Claudius Galenus it received a mighty impulse which extended to the fall of that vast empire; but with its disruption the art was again jeopardized,—only a faint spark remaining which floated over, and was fanned into life in the mystic land of the followers of the Koran. From Arabia it found its way into Africa, and from thence across the Mediterranean to Spain, where for a long time it made little progress. In Central Europe, now the center of medical science, no art, no science, no culture, found congenial recognition. The refinement of Hellas, of Alexandria, of Rome, was supplanted by the migrations and devastations of the Goths, the Vandals, the Franks and the Lombards. Brute force swayed the destiny of Europe, and for 600 years every possible obstacle was thrown in the way of rational medicine. What little there was of the healing was completely under ecclesiastical influence, and, at the time of Innocent III, in the twelfth century, no physician was allowed to visit a patient without first going to confessional. But an art so intimately associated with the happiness and welfare of humanity cannot always lie dormant even amidst the grossest ignorance and superstition, and with the establishment of the school of Salerus the physician again rose into prominence, and the teachings of Hippocrates, after many vicissitudes, received a new impulse, gathering forece and power, continually, down to our own time, resisting all antagonisms and adverse circumstances, triumphing over all opposition, and standing to-day, an enduring monument of humanity's highest, noblest and best impulses and sympathies.

